# MEDICAL STUDENT MOTIVATIONS TO STUDY GERIATRICS AS MEDICAL SPECIALTY IN ECUADOR: A QUALITATIVE APPROACH

**DOI:** 10.1093/geroni/igad104.2118

**Published:** 2023-12-21

**Authors:** Daniela Belen Sosa Cifuentes, Jonathan Guillemot

**Affiliations:** Universidad San Francisco de Quito, Quito, Pichincha, Ecuador; Universidad San Francisco de Quito, Quito, Pichincha, Ecuador

## Abstract

Due to a lack of interest in becoming geriatricians in the medical student community and despite studies showing geriatrics as one of the most fulfilling medical specialties, Ecuador, like many countries globally, lacks geriatricians. Although there are insufficient well-established institutions training geriatricians, the issue essentially lies in the lack of student vocations. We conducted a qualitative and participatory study to identify and describe the motivations and barriers associated with medical students’ interests in becoming geriatricians in Quito, Ecuador. Qualitative interviews between medical students after interviewing technique training were conducted. Audio recordings were transcribed and coded, and then analyzed for patterns. Thirty-two students were interviewed, none of which considered geriatrics as their first medical specialty option, and two of which considered the possibility of such a choice, but not as a first option. Among the most significant patterns associated with disinterest in geriatrics was the lack of exposure as well as a general ignorance of the lives of older adults beyond direct relatives. While most participants recognized the fundamental importance of the specialty, barriers appearing unsurmountable emerged: patterns of gerontophobia as well as thanatophobia were strong hurdles, combined with the perception of an emotional toll associated with the care of older adults. This joined with the view that physicians could not be fulfilling their purpose of “saving lives” in the context of geriatrics. As the Global South ages, universities must improve student exposure to older adults and the professions associated with their care as a first step towards promoting new vocations.

